# SCF^Cyclin F^-dependent degradation of CDC6 suppresses DNA re-replication

**DOI:** 10.1038/ncomms10530

**Published:** 2016-01-28

**Authors:** David Walter, Saskia Hoffmann, Eirini-Stavroula Komseli, Juri Rappsilber, Vassilis Gorgoulis, Claus Storgaard Sørensen

**Affiliations:** 1 Biotech Research and Innovation Centre (BRIC), University of Copenhagen, Ole Maaløes Vej 5, Copenhagen N 2200, Denmark; 2 Department of Histology and Embryology, School of Medicine, University of Athens, Athens GR-11527, Greece; 3 Wellcome Trust Centre for Cell Biology, University of Edinburgh, Michael Swann Building, Kings Buildings, Max Born Crescent, Edinburgh EH9 3BF, Scotland; 4 Department of Bioanalytics, Institute of Biotechnology, Technische Universität Berlin, Berlin 13355, Germany; 5 Faculty Institute of Cancer Sciences, University of Manchester, Manchester Academic Health Science Centre, Manchester, UK

## Abstract

Maintenance of genome stability requires that DNA is replicated precisely once per cell cycle. This is believed to be achieved by limiting replication origin licensing and thereby restricting the firing of each replication origin to once per cell cycle. CDC6 is essential for eukaryotic replication origin licensing, however, it is poorly understood how CDC6 activity is constrained in higher eukaryotes. Here we report that the SCF^Cyclin F^ ubiquitin ligase complex prevents DNA re-replication by targeting CDC6 for proteasomal degradation late in the cell cycle. We show that CDC6 and Cyclin F interact through defined sequence motifs that promote CDC6 ubiquitylation and degradation. Absence of Cyclin F or expression of a stable mutant of CDC6 promotes re-replication and genome instability in cells lacking the CDT1 inhibitor Geminin. Together, our work reveals a novel SCF^Cyclin F^-mediated mechanism required for precise once per cell cycle replication.

## Abstract (web summary)

To ensure genome stability, cells need to restrict DNA replication to once per cell cycle. Here the authors show that Cyclin F interacts with and targets the licensing factor CDC6 for degradation, preventing re-firing of replication origins.

## Graphical abstract

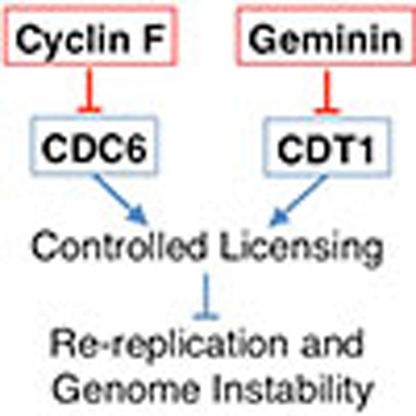

To ensure that chromosomal DNA is precisely duplicated and no sections of DNA are re-replicated, eukaryotic replication origins must fire no more than once in a single cell cycle^1^,^2^,^3^,^4^. To limit firing of origins to once per cell cycle, the two steps of replication initiation—origin licensing and origin firing—are temporally separated: first, CDC6 and CDT1 collaborate from late mitosis to late G1 phase to load complexes of the minichromosome maintenance 2–7 proteins at replication origins to form pre-replicative complexes (pre-RCs)^5^,^6^,^7^. Second, two types of protein kinases, cell division cycle 7 (CDC7) and the S phase cyclin-dependent kinases (CDKs) convert pre-RCs into bidirectional replisomes at each origin during S phase^8^,^9^,^10^. To avoid re-replication, it is crucially important to suppress licensing of newly replicated DNA until cells are in late phases of mitosis. Mechanisms that prevent re-licensing of replicated DNA include the degradation of the licensing protein CDT1 (refs 1, 2) as well as inhibition of CDT1 by Geminin^11^,^12^,^13^,^14^. Inhibition of CDC6, however, is poorly understood. CDC6 is phosphorylated by CDKs during S-phase and CDK-dependent phosphorylation of CDC6 beyond G1 can trigger nuclear export of exogenous CDC6 (refs 15, 16, 17). However, endogenous CDC6 is predominantly nuclear throughout the cell cycle^18^,^19^,^20^ and only a small fraction of endogenous CDC6 is exported from the nucleus^18^,^19^,^20^. Thus, mechanisms underlying control of CDC6 function beyond G1 phase are still elusive. Here we show that CDC6 is targeted for proteasomal degradation late in the cell cycle by the SCF^Cyclin F^ ubiquitin ligase complex. We show that CDC6 and Cyclin F interact through defined sequence motifs that promote CDC6 ubiquitylation and degradation. Absence of Cyclin F or expression of a stable mutant of CDC6 promotes re-replication and genome instability in cells lacking the CDT1 inhibitor Geminin. Together, our work reveals a novel SCF^Cyclin F^-mediated mechanism required for precise once per cell cycle replication.

## Results

### CDC6 and cyclin F interact through defined molecular motifs

**Figure 1:**
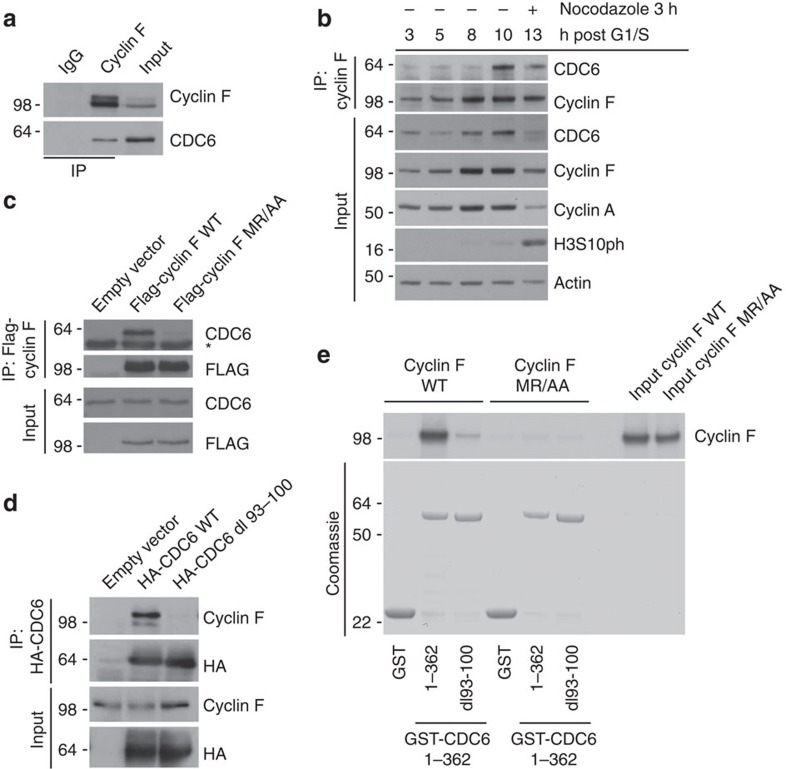
CDC6 and Cyclin F interact through defined molecular motifs. (**a**) Whole-cell extracts of U2OS cells were generated, lysates were immunoprecipitated (IP) with anti-Cyclin F antibody and IPs were analysed by immunoblotting as indicated. (**b**) U2OS cells were synchronized at G1/S by using a double-thymidine block before release into fresh medium. Whole-cell extracts were generated at the indicated time points, lysates were IP with anti-Cyclin F antibody and IPs were analysed by immunoblotting as indicated. (**c**) HEK-293T cells were transfected with an empty vector, FLAG-tagged wild-type Cyclin F (WT) or FLAG-tagged mutant Cyclin F(MR/AA). Whole-cell extracts were IP with anti-FLAG resin, and IPs were analysed by immunoblotting as indicated. *Notes a cross reacting band in the CDC6 immunoblot. (**d**) HEK-293T cells were transfected with an empty vector, HA-tagged wild-type CDC6 (WT) or HA-tagged mutant CDC6 (dl 93–100). Whole-cell extracts were IP with anti-HA resin, and IPs were analysed by immunoblotting as indicated. (**e**) The interaction between recombinant WT and dl 93–100 mutant GST-CDC6 fragments (amino acids 1–362) and *in-vitro* translated and S35-methionine-labelled WT and MR/AA mutant Cyclin F was tested. GST alone was used as a negative control. The GST-fusion proteins were precipitated by glutathione-agarose affinity chromatography and the interaction with the *in vitro* translated S35-labelled proteins was visualized by autoradiography.

It is not well understood how CDC6 activity is suppressed in cell cycle stages beyond G1 phase, which are the phases where re-replication is an obvious threat to genome integrity. We hypothesized that the SCF^Cyclin F^ ubiquitin ligase could be central in regulating CDC6 activity because we uncovered a very marked interaction between Cyclin F and CDC6 in a mass spectrometry screen for Cyclin F interactors (Supplementary Fig. 1a). Cyclin F is the founding member of the family of F-box proteins, which are evolutionarily conserved substrate recognition subunits of SCF(Skp1-Cul1-F-box protein) ubiquitin ligase complexes and mediate the proteolysis of eukaryotic proteins^21^,^22^. Cyclin F was previously shown to regulate deoxyribonucleotide triphosphate production, centrosome duplication and spindle formation through targeted degradation of SCF^Cyclin F^ substrates, that is, NUSAP1, RRM2 and CP110 (refs 23, 24, 25). Furthermore, we recently uncovered a novel role for Cyclin F in suppression of B-Myb through direct protein interaction^26^. As expected from the known function of Cyclin F, the mass spectrometry analysis also revealed the presence of many peptides corresponding to the SCF^Cyclin F^ subunits Skp1 and Cul1 (Supplementary Fig. 1a). To confirm the association between CDC6 and Cyclin F, we immunoprecipitated endogenous Cyclin F and detected an interaction with endogenous CDC6 (Fig. 1a). This interaction occurs predominantly during late cell cycle stages, that is, G2 and M phase as shown by co-immunoprecipitation analysis of endogenous Cyclin F in synchronized U2OS cells (Fig. 1b). Because both Cyclin F and CDC6 localize predominantly but not exclusively to the nucleus^15^,^25^, we asked where the two proteins could interact. Cells stably expressing V5-tagged CDC6 were co-stained with antibodies to Cyclin F and V5 tag. Both Cyclin F- and V5-tagged CDC6 were nuclear during G2 phase of the cell cycle (Supplementary Fig. 1a). These results together with the co-immunoprecipitation experiments suggest that Cyclin F and CDC6 interact in the nucleus.

We next investigated the interaction between Cyclin F and CDC6. Cyclin F has a Cyclin box domain, which contains a hydrophobic patch motif^22^,^25^. In Cyclin F, this hydrophobic patch with the amino-acid sequence MRYIL (Met-Arg-Tyr-Ile-Leu) associates with the cyclin-binding motif (Cy motif) Arginine-X-Leucine in its substrates^24^,^25^. We asked whether the interaction between Cyclin F and CDC6 is dependent on the Cyclin box domain in Cyclin F and found that Cyclin F containing a mutation in the hydrophobic patch (MR/AA) failed to co-immunoprecipitate endogenous CDC6 (Fig. 1c). Notably, co-immunoprecipitation analysis of mutant Cyclin F MR/AA revealed binding with the SCF(Cyclin F) subunit Cul1 (Supplementary Fig. 1d), suggesting that overall integrity of mutant Cyclin F MR/AA is not impaired. Further, we investigated whether the interaction between Cyclin F and CDC6 depends on the conserved Cy motif in CDC6. CDC6 contains a Cy motif between amino acids 93 and 100 (ref. 15). Deletion of amino acids 93–100 (dl 93–100) containing the Cy-motif abolishes the interaction between CDC6 and Cyclin F (Fig. 1d) but not between CDC6 and its known interaction partner CDH1 (refs 27, 28; Supplementary Fig. 1c), suggesting that the complex formation between Cyclin F and CDC6 depends on the Cy-motif in CDC6 and that the integrity of CDC6 dl93–100 is not impaired. Next, we analysed whether Cyclin F and CDC6 are interacting directly and if so, whether the Cy-motif in CDC6 and the Cyclin box domain in Cyclin F are required for this interaction. We co-incubated purified recombinant WT and dl 93–100 mutant glutathione *S-*transferase (GST)-tagged CDC6 N-terminal fragments (GST-CDC6 1–362 WT and GST-CDC6 1–362 dl93–100), respectively, with either *in vitro* translated 35S-labelled WT or MR/AA mutant Cyclin F (Fig. 1e). We found that the WT but not MR/AA mutant Cyclin F interacts with GST-CDC6 1–362 WT (Fig. 1e). Further, we found that this interaction depends on amino acids 93 to 100 in CDC6 (Fig. 1e). Together, these data suggest that CDC6 directly interacts with Cyclin F via the Cy-motif in CDC6 and the Cyclin box domain in Cyclin F.

### CDC6 is targeted for degradation by SCF^Cyclin F^

**Figure 2:**
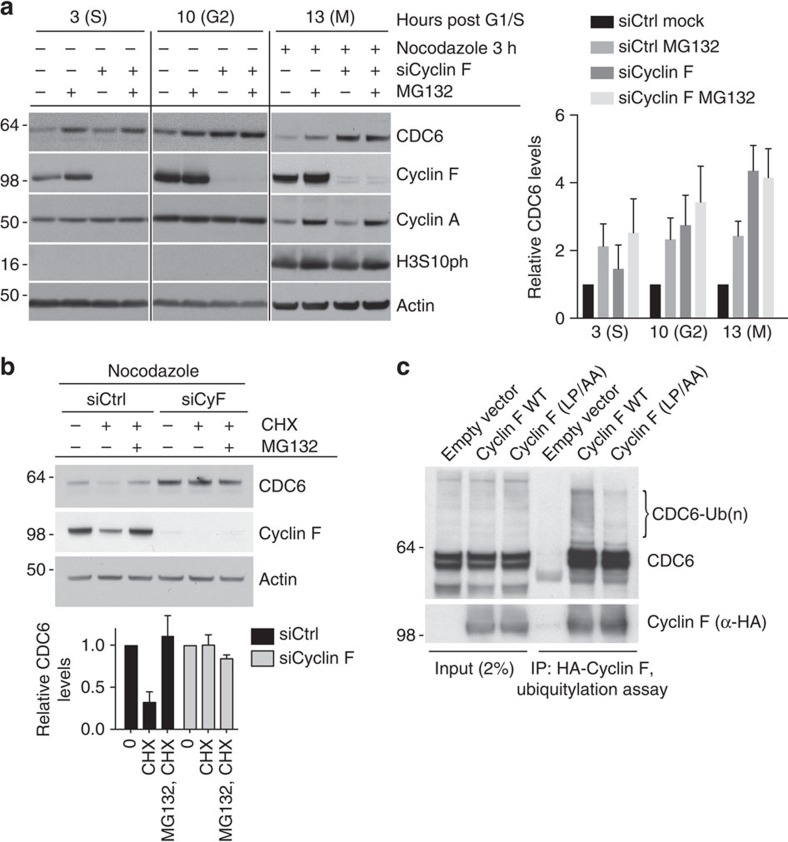
CDC6 is targeted for ubiquitylation and degradation by SCF^Cyclin F^. (**a**) U2OS cells were synchronized at G1/S by using a double-thymidine block. Transfection of siRNAs was performed in the first thymidine block as indicated. Cells were released and treated with MG132 for 90 min as indicated and whole-cell extracts were analysed by immunoblotting as indicated. Nocodazole was added for 3 h as indicated to prevent cells from entering the next G1 phase. Quantification of immunoblots of CDC6 is indicated. Individual lanes were quantified using ImageJ software and normalized to Actin loading control. For each time-point, the protein level of CDC6 transfected with control siRNA (siCtrl) in the absence of MG132 was arbitrary set to 1. Error bars represent s.d. (*n*=3). (**b**) U2OS cells were transfected with control (siCtrl) or Cyclin F (siCycl F) siRNA. After 48 h, cells were treated with nocodazole for an additional 5 h and the protein stability in cells obtained by mitotic shake-off was assessed by CHX and MG132 treatment for 90 min and immunoblotting as indicated. Protein stability of CDC6 was assessed and relative CDC6 abundance was determined by densitometry and normalized to actin with the amount of CDC6 in the first and fourth line, respectively, arbitrarily set to 1. Error bars represent s.d. (*n*=3). (**c**) HEK-293T cells were transfected with an empty vector, HA-tagged wild-type Cyclin F (WT), or HA-tagged mutant Cyclin F(LP/AA). Whole-cell extracts were immunoprecipitated (IP) with anti-HA resin and used in a ubiquitylation assay.

To test whether Cyclin F could regulate protein stability of CDC6 through proteasomal degradation, we analysed CDC6 protein levels throughout the cell cycle in the presence and absence of Cyclin F. Endogenous CDC6 is unstable and it accumulated after treatment of cells with the proteasomal inhibitor MG132 during S (*t*=3), G2 (*t*=10) and mitosis (Fig. 2a). Importantly, Cyclin F depletion led to accumulation of CDC6 in G2 and M phase but had little impact on CDC6 levels in S phase (Fig. 2a). This indicates that proteasomal degradation of CDC6 in G2/M phase cells is dependent on Cyclin F. Consistently, depletion of Cyclin F in U2OS cells with several different short interfering RNAs (siRNAs) led to increased CDC6 protein levels during mitosis (Supplementary Fig. 2a). To confirm that CDC6 is regulated by proteasomal degradation in a Cyclin F-dependent manner, we analysed CDC6 protein levels during mitosis in the presence of cycloheximide (CHX). CHX allows analysis of protein turnover by blocking new protein synthesis through translation inhibition. Indeed, we found that both depleting Cyclin F as well as inhibiting the proteasome with MG132 caused stabilization of CDC6 during mitosis after CHX addition (Fig. 2b). Consistent with the role of SCF-mediated control of CDC6, treatment of cells with the SCF complex E3 ligase inhibitor MLN4924 caused stabilization of CDC6, which was marked after CHX addition during mitosis (Supplementary Fig. 2b). The anaphase-promoting complex (APC) targets CDC6 for degradation during G1 phase^28^, but has no impact on CDC6 stability in mitosis as evidenced by stability measurement upon CDH1 depletion, the substrate adaptor protein of APC (Supplementary Fig. 2c). Notably, immunoprecipitated wild-type (WT) Cyclin F, but not F-box mutant Cyclin F(LP/AA)^25^, promoted the *in vitro* ubiquitylation of co-immunoprecipated CDC6 (Fig. 2c). Together, these results suggest that Cyclin F mediates the degradation of CDC6 during G2 and M phase of the cell cycle by forming an active SCF ubiquitin ligase complex.

**Figure 3:**
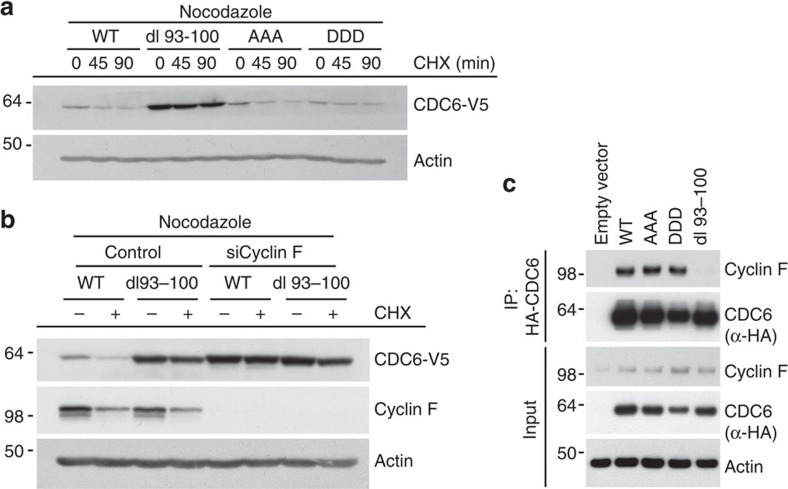
CDC6 recognition by Cyclin F is independent of CDK phosphorylation. (**a**) U2OS cells stably expressing either V5-tagged wild-type CDC6 (WT) or V5-tagged mutant Cdc6 (AAA, DDD or dl 93–100, respectively) were treated with nocodazole for 5 h and the protein stability of WT and mutant Cdc6 in cells obtained by mitotic shake-off was assessed by CHX treatment and immunoblotting. (**b**) U2OS cells stably expressing either V5-tagged wild-type CDC6 (WT) or V5-tagged mutant CDC6 (dl 93–100) were transfected with control or Cyclin F siRNAs. After 48 h, cells were treated with nocodazole for an additional 5 h and the protein stability of WT and mutant CDC6 in cells obtained by mitotic shake-off was assessed by CHX treatment for 90 min and immunoblotting. (**c**) U2OS cells were transfected with empty vector (EV), HA-tagged wild-type CDC6 (WT) or HA-tagged mutant CDC6 (AAA, DDD or dl 93–100, respectively) and total cell extracts were subjected to immunoprecipitation with anti-HA resin. Immunoprecipitates were analysed by immunoblotting as indicated.

The amino terminus of CDC6 contains three CDK phosphorylation sites, that is, serine residues S54, S74 and S106 (ref. 15). These phosphorylation sites appear to be the major functional CDK sites in CDC6, as mutation of these abolishes phosphorylation of CDC6 *in vivo* and *in vitro*^15^,^17^. Phosphorylation of yeast Cdc6p by S and M phase CDKs targets Cdc6p for SCF-dependent ubiquitylation and subsequent degradation^29^. In contrast, human CDC6 phosphorylated by CDKs is prone to nuclear export^15^,^16^,^17^ and was shown to be protected from APC-dependent ubiquitylation and subsequent degradation^27^. To investigate whether CDK-mediated phosphorylation of CDC6 regulates Cyclin F-mediated degradation of CDC6, we asked whether protein stability of CDC6 during mitosis is modulated by CDK phosphorylation. We analysed protein stability of mutant CDC6 where the three known CDK-phosphorylated serine residues S54, S74 and S106 were replaced with either alanine (AAA) to mimic the unphosphorylated state or aspartic acid (DDD) to mimic the phosphorylated state. Protein stability of AAA or DDD CDC6 was not altered after CHX addition during mitosis when compared with WT CDC6 (Fig. 3a). In contrast, the dl 93–100 mutant was more stable than the WT CDC6 protein after CHX addition (Fig. 3a). Similarly, dl 93–100 CDC6 stably expressed in human TIG-3 fibroblasts was markedly increased during mitosis when compared with WT, AAA and DDD CDC6 (Supplementary Fig. 3a). Notably, the increased stability of dl93–100 mutant CDC6 was not caused by a lack of APC-dependent degradation of CDC6, as the stability of CDC6 mutated in the APC recognition motifs RKRL and KEN at residues 56–59 and 81–83 (mutated to ‘AKRA' and ‘AAA', respectively) was comparable to WT CDC6 during mitosis (Supplementary Fig. 3b). Notably, WT and dl93–100 mutant CDC6 exhibited indistinguishable cellular localization throughout the cell cycle (Supplementary Fig. 3c), suggesting that the change in protein stability is not a consequence of different localization of WT and dl93–100 mutant CDC6, respectively. Furthermore, reduction of Cyclin F by siRNA stabilized WT CDC6 but had no effect on the already stabilized dl 93–100 mutant (Fig. 3b). Finally, Cyclin F could be detected in haemagglutinin (HA) immunoprecipitates with either WT, AAA or DDD HA-CDC6 but not with dl 93–100 HA-CDC6 (Fig. 3c). Thus, WT, as well as AAA and DDD mutant CDC6, are regulated by SCF^Cyclin F^, but the dl 93–100 mutant is resistant to Cyclin F-mediated degradation because of its inability to interact with Cyclin F.

### Cyclin F suppresses genome instability

**Figure 4:**
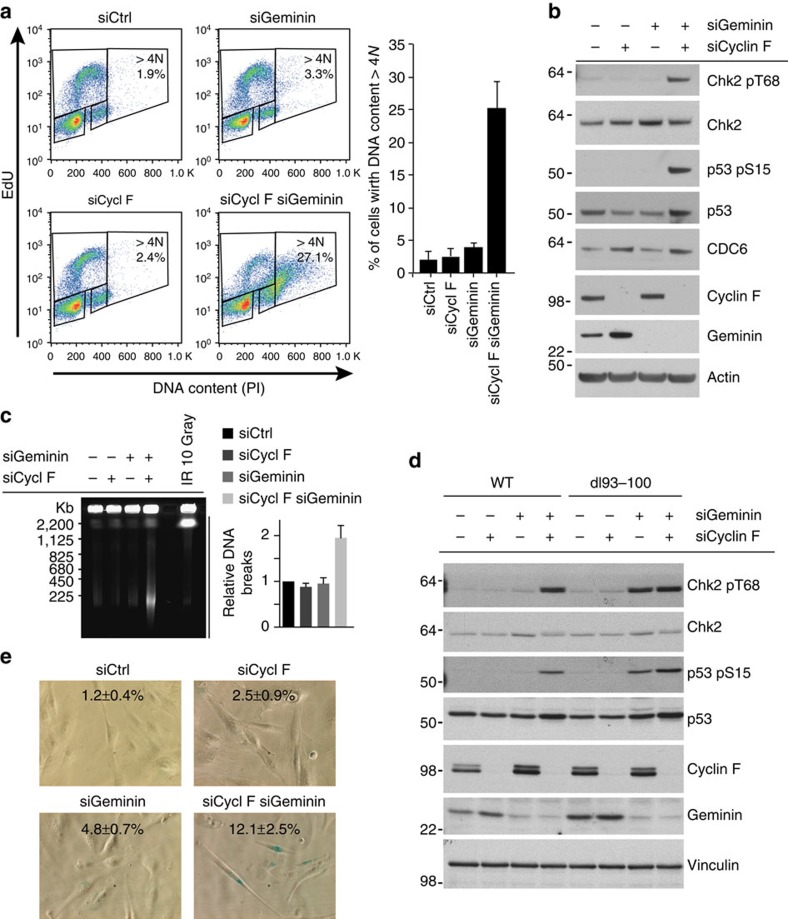
Cyclin F suppresses genome instability. (**a**) U2OS cells were first transfected with siRNA targeting Cyclin F as indicated. After 24 h, transfection with siRNA targeting Geminin was performed as indicated. Cells were collected 72 h after the first transfection after pulse-labelling with EdU for 1 h. Cells were subjected to Click-it reaction and propidium iodide (PI) staining before analysis by flow cytometry. The graph shows the results of three independent experiments. Error bars represent s.d. (*n*=3). (**b**) The experiment was performed as in **a**, and whole-cell extracts were analysed by immunoblotting as indicated. (**c**) The experiment was performed as in **a** and cells were subjected to PFGE analysis. Bands representing DNA breaks are indicated. The graph shows the quantification of three independent experiments where bands were quantified using ImageJ software and error bars represent s.d. (*n*=3). (**d**) U2OS cells stably expressing either V5-tagged wild-type CDC6 (WT) or V5-tagged mutant CDC6 (dl 93–100) were transfected with siRNA as described in **a**. Whole-cell extracts were analysed by immunoblotting as indicated. (**e**) TIG-3 cells were transfected every 3 days with the indicated siRNAs and senescence-associated β-galactosidase (SA-β-gal) staining was performed after 6 days of depletion. Means and s.d. of SA-β-gal-positive cells from three independent experiments are indicated.

Licensing of DNA replication origins by the assembly of pre-RCs is tightly regulated and requires the combined action of CDC6 and CDT1 (refs 5, 6, 7). Reduction of CDT1 activity by proteasomal degradation or by the CDT1 inhibitor Geminin have been described as mechanisms preventing re-replication by re-licensing of replicated DNA during late cell cycle stages^11^,^12^. We analysed whether Cyclin F-mediated degradation of CDC6 is preventing re-replication and analysed the DNA content in cells treated with siRNA targeting Cyclin F. Here, we observed limited re-replication and accumulation of cells with DNA content greater than 4 N in Cyclin F-depleted cells (Fig. 4a and Supplementary Fig. 4a,c). These results are consistent with data obtained in several systems, in which the overexpression of CDC6 is not sufficient to induce re-replication^15^,^28^,^30^,^31^. Furthermore, the data are consistent with the notion that CDC6 and CDT1 cooperate in licensing and that several overlapping mechanisms exist to prevent re-replication^3^. We therefore hypothesized that the degradation of CDC6 by Cyclin F cooperates with mechanisms that suppress CDT1 activity. Indeed, depletion of both Cyclin F and the CDT1 inhibitor Geminin caused substantial re-replication as manifested by a proportion of cells with DNA content greater than 4 N (Fig. 4a and Supplementary Fig. 4a,c). We further analysed DNA re-replication at intermediate times after siRNA-induced depletion of Cyclin F and/or Geminin (Supplementary Fig. 4b,c) and consistently found substantially more cells with DNA content >4 N in cells co-depleted for Cyclin F and Geminin as compared with Geminin depletion alone (Supplementary Fig. 4c), suggesting that Cyclin F is important for suppressing re-replication when CDT1 activity is high. Re-replication leads to genomic instability as evidenced by the formation of DNA double-strand breaks (DSBs), and these DNA lesions activate ataxia-telangiectasia mutated checkpoint pathways^32^. We therefore measured DNA DSB formation and ataxia-telangiectasia mutated-dependent DNA damage checkpoint activation in siRNA-treated cells. Depletion of Cyclin F or Geminin alone did not lead to marked DNA DSBs and DNA damage checkpoint activation (Fig. 4b and Supplementary Fig. 4d). However, combined depletion of both Cyclin F and Geminin lead to marked formation of DSBs and the activation of DNA damage checkpoints (Fig. 4b,c and Supplementary Fig. 4d). These data further highlight that Geminin and Cyclin F cooperate in maintaining genome integrity, through joint suppression of the major licensing factors CDC6 and CDT1. Notably, siRNA depletion of Geminin in cells expressing the dl93–100 mutant, but not WT CDC6, caused a marked activation of DNA damage checkpoint signalling and re-replication (Fig. 4d and Supplementary Fig. 5a). In comparison to previous reports^11^,^13^,^14^, we observed rather limited re-replication in Geminin-depleted cells. We hypothesized that the underlying reason for the observed difference may be a consequence of our experimental setup of the co-depletion experiment, where cells are first depleted with Cyclin F and control siRNA, respectively, and 24 h later with Geminin and control siRNA, respectively. To test whether this is indeed the case, we depleted Geminin in U2OS cells stably expressing V5-tagged LacZ, WT CDC6 or the stable dl93–100 mutant CDC6, respectively. Under these conditions, we found that Geminin depletion in cells expressing LacZ and WT CDC6 leads to re-replication similar to previous studies when Geminin was depleted in U2OS cells^11^,^14^ (Supplementary Fig. 5b). However, Geminin depletion in cells expressing the stable dl93–100 mutant CDC6 caused a dramatic increase in re-replication as compared with Geminin depletion in cells expressing LacZ and WT CDC6, respectively (Supplementary Fig. 5b). Together, these data suggest that Cyclin F prevents re-replication and genome instability by targeting CDC6 for degradation. We further analysed the biological significance of the checkpoint response in the absence of Cyclin F as observed in Fig. 4b–d. Cellular senescence is a tumorigenesis barrier imposed by DNA damage checkpoints^33^,^34^ and we therefore speculated that the absence of Cyclin F in cells with aberrant CDT1 activity would lead to cellular senescence. Indeed, we observed induction of cellular senescence in TIG-3 human fibroblasts depleted for Cyclin F and Geminin as evidenced by senescence-associated β-galactosidase activity (Fig. 4e).

### Cyclin F suppresses DNA re-replication during G2 phase

**Figure 5:**
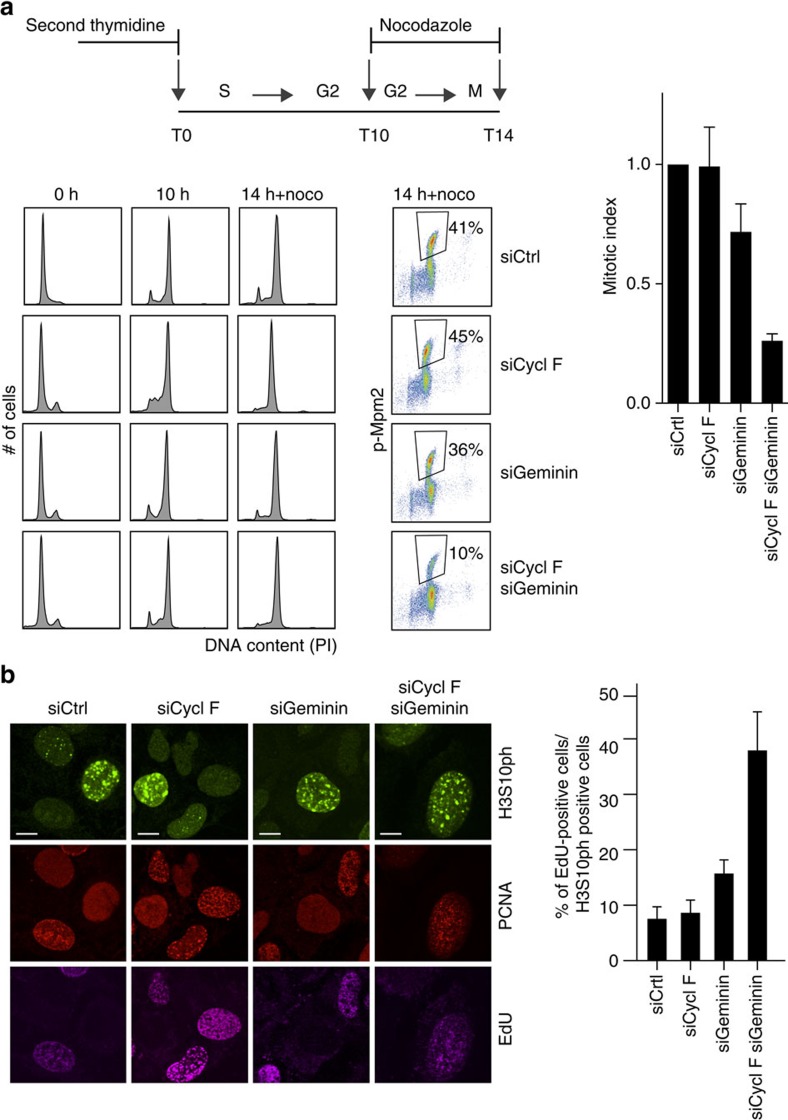
Cyclin F-mediated suppression of re-replication occurs during G2 phase. (**a**) Schematic shows experimental setup including a double thymidine synchronization and nocodazole treatment. U2OS cells were transfected with siRNAs targeting Cyclin F and Geminin in the first and second thymidine block, respectively. At the indicated time points, cells were given nocodazole for 4 h to trap cells in mitosis. Cells were collected at indicated time points and DNA content and mitotic MPM2 phosphorylation (p-MPM2) was monitored by flow cytometric analysis as indicated. The bar chart shows the mitotic index analysed by p-MPM2 and normalized to control depletion (siCtrl). Error bars represent s.d. (*n*=3). (**b**) U2OS cells were first transfected with siRNA targeting Cyclin F as indicated. After 24 h, transfection with siRNA targeting Geminin was performed as indicated. Before harvesting 60 h after the first transfection, cells were pulse-labelled with EdU for 45 min. Cells were fixed and subjected to Click-it reaction, PCNA and histone H3S10ph staining before analysis by immunofluorescence. Scale bar, 10 μm. The bar chart shows the percentage of H3S10ph-positive cells that have EdU incorporated. Error bars represent s.d. (*n*=3).

Next, we hypothesized that Cyclin F-mediated degradation of CDC6 is critical to limit licensing specifically during late cell cycle stages, that is, G2 and M phase, when Cyclin F associates with and targets CDC6 for degradation (Figs 1b and 2a,b). Notably, we found that cells co-depleted for Cyclin F and Geminin completed an apparently normal S phase (Fig. 5a and Supplementary Fig. 6a,b) and exhibited no obvious signs of DNA replication stress and DNA damage such as phosphorylation of CHK1 at S317 and RPA at S4/S8 (Supplementary Fig. 6b,c), but underwent cell cycle delay at the G2-M boundary (Fig. 5a). Similarly, siRNA depletion of Geminin in cells expressing dl93–100 mutant but not WT CDC6 imposed a cell cycle delay at the G2-M boundary (Supplementary Fig. 5c). Notably, cells with DNA content >4 N started to appear after completing an apparently normal S phase in the absence of Cyclin F and Geminin 15 h after a G1/S release (Supplementary Fig. 6a). We therefore reasoned that inhibition of CDC6 by Cyclin F prevents licensing and re-replication during G2 and early M phase and that the observed cell cycle arrest in cells co-depleted for Cyclin F and Geminin is caused by re-replication in G2 cells. To test whether replication in cells co-depleted for Cyclin F and Geminin is indeed occurring during G2 phase of the cell cycle, we analysed aberrant DNA replication during late cell cycle stages. Cells were pulse labelled with the thymidine analogue 5-ethynyl-2'-deoxyuridine (EdU) for 45 min immediately before cell fixation, and late G2 and mitotic cells were analysed for EdU incorporation by co-staining cells for phosphorylated histone H3 at serine 10 (H3S10p), a marker for late G2 and M phase. Most control-, Cyclin F- and Geminin-depleted H3S10p-positive cells were EdU negative (Fig. 5b), as expected to be the case in cells where DNA replication was completed before the EdU pulse, that is, at least 45 min before the onset of H3S10 phosphorylation in late G2. However, a substantial amount of cells co-depleted for Cyclin F and Geminin that were positive for H3S10 phosphorylation were also replicating as evidenced by positive EdU incorporation (Fig. 5b). Thus, our data suggest that Cyclin F and Geminin suppress re-replication during G2 phase, a cell cycle stage where available CDK and CDC7 kinase activities can support firing of inappropriately licensed origins.

## Discussion

To ensure replication happens once and only once during the cell cycle, the two steps of replication initiation, origin licensing and firing, are temporarily separated. If inappropriate re-licensing of replication origins occurs from S phase to mitosis, origins may fire more than once within a single cell cycle, which can result in re-replication of DNA. Several overlapping mechanisms prevent re-licensing^1^,^2^,^11^,^12^,^13^,^14^, however, the regulation of the key licensing protein CDC6 was so far poorly understood in this process. The results presented here reveal that the E3-ligase SCF^Cyclin F^ restrains CDC6 activity to suppress re-replication. Cyclin F accumulates in late stages of the cell cycle^24^,^25^, and our data uncover a pronounced interaction with CDC6 at this stage mediated via a specific Cyclin-binding motif. This promotes SCF^Cyclin F^-dependent degradation of CDC6 thereby safeguarding chromosomal DNA by securing that no sections of DNA are over-replicated. In the absence of Cyclin F, cells critically rely on Geminin and control of CDT1 to suppress re-replication causing genome instability and checkpoint activation. Consistently, the absence of Cyclin F can induce cellular senescence, a tumorigenesis barrier imposed by DNA damage checkpoints. The overlapping mechanisms acting to prevent re-replication ensure that lack of one regulatory pathway is not sufficient to induce re-replication. In agreement with this notion, we found only little induction of re-replication in response to Cyclin F depletion. Co-depletion of Cyclin F together with Geminin, however, induces substantial re-replication, suggesting that the degradation of CDC6 by Cyclin F cooperates with mechanisms that suppress CDT1 activity. It was previously reported that Geminin ablation in itself promotes re-replication^11^,^13^,^14^. Here, we observe only little re-replication in Geminin-depleted cells, which is likely due to the difference in our experimental setup of the co-depletion experiment, where Cyclin F is depleted 24 h before Geminin. In any case, absence of Cyclin F strongly promotes re-replication in Geminin knockdown cells.

In mammalian cells, CDK phosphorylation of pre-RC components has a direct role in blocking licensing (reviewed in ref. 3). Specifically, CDK-phosphorylated CDT1 and ORC1 are targeted for ubiquitin-mediated degradation by CRL1^Skp2^ (refs 35, 36) and Orc2 chromatin binding is inhibited upon CDK phosphorylation^37^,^38^. CDC6 is also phosphorylated by CDKs but whether this inhibits pre-RC assembly in higher eukaryotes is still unclear. CDK-dependent phosphorylation of CDC6 beyond G1 can trigger nuclear export of exogenous CDC6 (refs 15, 16, 17). However, endogenous CDC6 is predominantly nuclear throughout the cell cycle^18^,^19^,^20^ and only small fraction of endogenous CDC6 is exported from the nucleus^18^,^19^,^20^. In this study, we have not observed a role for CDKs in Cyclin F mediated CDC6 inhibition. Mutations in CDC6 where all three known CDK-phosphorylated serines were replaced with either alanine (AAA) to mimic the unphosphorylated state or aspartic acid (DDD) to mimic the phosphorylated state did not impact the Cyclin F-CDC6 pathway. However, we cannot exclude that novel CDK sites or additional post-translational modifications in CDC6 impact the Cyclin F-CDC6 pathway. In addition, it is possible that the interaction between CDC6 and Cyclin F counteracts the ability of other cyclins, such as Cyclin A or Cyclin B, to interact with CDC6 through its Cy-motif. The functional and regulatory roles of these potentially competitive interactions with CDC6 is an interesting topic for futures studies. Moreover, our data support a role for neddylation in CDC6 turnover^39^, which supports a function of the cullin E3 ligase SCF^Cyclin F^ in regulating CDC6 stability. Cullin E3 ligases such as SCF^Cyclin F^ can be inhibited by MLN4924 (ref. 40), an inhibitor of cullin neddylation and a potent antiproliferative agent that has completed phase I trials (http:// www.clinicaltrials.gov). We found that inhibition of cullin neddylation by MLN4924 stabilized CDC6 late in the cell cycle similar to Cyclin F depletion. MLN4924 treatment of cells induces massive re-replication^11^,^41^, which was reported to be mediated through inhibition of CDT1 degradation^41^. Our new findings suggest that disruption of the degradation of CDC6 contributes to MLN4924 effectiveness.

Cyclin F is emerging as a new factor controlling several key cell cycle processes late in the cell cycle including deoxyribonucleotide triphosphate production, centrosome duplication, spindle formation and DNA damage checkpoint maintenance through NUSAP1, RRM2, CP110 and B-Myb^23^,^24^,^25^,^26^. In all cases, Cyclin F operates to maintain homeostasis through the hydrophobic patch in its Cyclin domain. This study adds an important new biological process to the repertoire of Cyclin F actions operating by the hydrophobic patch-dependent degradation of CDC6 to safeguard that chromosomal DNA is precisely duplicated.

## Methods

### Cell culture and cell cycle synchronization and chemicals

U2OS, TIG-3-hTERT and HEK-293T cells were obtained from American Type Culture Collection and maintained in DMEM containing 10% fetal bovine serum. Stable cell lines expressing CDC6-V5 constructs were obtained by transducing U2OS and TIG-3-hTERT cells, respectively, with pLenti6/UbC/CDC6-V5 constructs and selecting transduced cells with 5 μg ml^−1^ Blasticidin. U2OS cell lines expressing Flag-HA-tagged Cyclin F were described previously^26^. For synchronization at G1/S, U2OS cells were cultured in the presence of 2 mM thymidine (Sigma) for 20 h, washed three times with PBS and cultured in fresh medium without thymidine for 10 h. After another 17 h in thymidine, cells were washed three times with PBS and cultured in fresh medium. To trap cells in prometaphase, U2OS cells were cultured in the presence of nocodazole (40 ng ml^−1^) for 5 h, before mitotic cells were obtained by mitotic shake-off. The following additional drugs were used: 10 μM MG132 (Sigma) and 25 μg ml^−1^ CHX (Sigma).

### Plasmids and siRNA

pCMVHA-CDC6, pCMVHA-CDC6 dl 93–100, pCMVhCDC6 AAA and pCMVhCDC6 DDD plasmids^15^ encoding N-terminal HA-tagged human WT and mutant CDC6 were gifts from Dr Kristian Helin. To create pLenti6/UbC/CDC6-V5 constructs, sequences encoding for WT and mutant CDC6 were amplified by PCR and cloned into pLenti6/UbC/V5-DEST (Invitrogen) according to the manufacturer's instructions. To create truncated CDC6 expression constructs, sequences encoding for WT and mutant CDC6 were amplified by PCR and cloned into a pCR8/GW/TOPO vector (Invitrogen). The following primers were used: CDC6: 5′-ATGCCTCAAACCCGATCC-3′, 1086 CDC6: 5′-TTATACCTGATTAAGTCGATCTTGCAA-3′. The entry vectors were used to generate pGEX-6P-1 expression vector using the Gateway LR Clonase II enzyme mix (Invitrogen) according to the manufacturer's protocol.

HEK-293T cells were transfected with plasmid DNA using the calcium phosphate method, as described^26^. U2OS were transfected with plasmid DNA using FuGENE 6 (Promega) according to the manufacturer's instruction.

siRNA transfections were performed with 50 nM siRNA duplexes using Lipofectamine RNAiMAX (Invitrogen), according to the manufacturer's instructions. The siRNA sequences used for knockdown are (5′→3′): Cyclin F #1 (GGAGGACAGAUGUGUCAGA), Cyclin F #2 (GCGCAGCUGUCUUUAGCCA), Cyclin F #3 (UAGCCUACCUCUACAAUGA), Geminin (AACUUCCAGCCCUGGGGUUAU), and p53 (GCAGUCAGAUCCUAGCGUC).

### Flow cytometry

To prepare cells for flow cytometry, cells were fixed in 70% ethanol. The cells were stained with Anti-phospho-Ser/Thr-Pro MPM-2 Antibody (1:200, Millipore) for 1 h followed by 1 h incubation with Alexa Fluor-488 anti-mouse (1:1,000) secondary antibodies. DNA was stained with 0.1 mg ml^−1^ propidium iodide containing RNase (20 μg ml^−1^) for 30 min at 37 °C. For EdU labelling, cells were incubated with 10 μM EdU for 1 h before harvest. Click-it reaction was performed according to the manufacturer's description (Invitrogen). Flow cytometry was performed on a FACS Calibur (BD Biosciences) using CellQuest Pro software (Becton Dickinson).

### Immunoblotting and antibodies

Cells were lysed on ice in cold EBC-buffer (150 mM NaCl; 50 mM Tris, pH 7.4; 1 mM EDTA; 0.5% NP-40/Igepal) containing protease inhibitors (1% aprotinin, 5 μg ml^−1^ leupeptin, 1 mM phenylmethylsulphonyl fluoride (PMSF)), phosphatase inhibitors (50 mM NaF; β-glycerophosphate; 0.5 μM Calyculin A) and 1 mM dithiothreitol (DTT). The lysates were sonicated using a digital sonifier (102C CE Converter; Branson). Proteins were separated by SDS–PAGE and transferred to a nitrocellulose membrane. Blocking and blotting with primary antibodies were performed in PBS-T supplemented with 5% skimmed milk powder. Proteins were visualized on films using secondary horseradish peroxidase-conjugated antibodies (1:10,000; Vector Laboratories) and enhanced chemiluminescence reagent (ECL) (GE Healthcare). Films were developed using an X-ray machine (Valsoe; Ferrania Technologies). The following commercial rabbit antibodies were used in this study: Cyclin F (sc-952, SCBT, 1/4,000 dilution), phospho-CHK2-T68 (no. 2661, Cell Signaling, 1/1,000 dilution), Geminin (A300–935A, Bethyl Labs, 1/500 dilution) and p53-S15 (no. 9284, Cell Signaling, 1/1,000 dilution), Phospho-RPA32 S4/S8 (A300-245A, Bethyl Labs, 1/1,000 dilution), Phospho-Chk1 S317 (no. 2344, Cell Signaling, 1/1,500 dilution). The following commercial mouse antibodies were used in this study: Actin (mAB1501, Chemicon Int,, 1/20,000 dilution), V5 (NB100–62264, Novus, 1/500 dilution), Vinculin (V9131, Sigma, 1/100,000 dilution), Flag (F3165, Sigma, 1/5,000 dilution), HA (MMS101P, Covance, ½,000 dilution), Anti-phospho-Ser/Thr-Pro MPM-2 Antibody (05–368, Milipore, 1/1,000 dilution), CHK2 (DCS-270, SCBT, 1/1,000 dilution) and p53 (DO-1, supernatant, 1/100 dilution). Mouse antibodies raised against CDC6 were described previously^15^ and used in a dilution of 1:5. Supplementary Fig. 7 contains uncropped scans of the immunoblots.

### Immunofluorescence microscopy

For Cyclin F and V5 co-staining, cells grown on coverslips were washed with PBS, fixed with formaldehyde 4% for 10 min, permeabilized with PBS containing 0.3% Triton X-100 for 10 min and blocked for 1 h in PBS containing 0.1% Triton X-100, 3% BSA before incubation with primary antibodies Cyclin F (sc-952, SCBT, 1/500 dilution), V5 (NB100–62264, Novus, 1/500 dilution), Phospho-RPA32 S4/S8 (A300-245A, Bethyl Labs, 1/1,000 dilution). For proliferating-cell nuclear antigen (PCNA) and H3S10ph co-staining, cells grown on coverslips were washed with PBS, then extracted for 1 min in cold pre-extraction buffer (0.5% Triton X-100, 20 mM HEPES, pH 7.5, 300 mM sucrose, 50 mM NaCl and 3 mM MgCl_2_), washed with PBS and fixed for 15 min in methanol at −20 °C. After additional washing in PBS cell were incubated with PCNA (Santa Cruz #SC-56, 1/1,000 dilution) and H3S10ph (Millipore, 06–570, 1/1,000 dilution) antibodies. Secondary antibodies were from donkey and conjugated with Alexa Fluor fluorochromes (Invitrogen, 1/1,000 dilution). EdU was detected using Click-IT Alexa Fluor 647 azide (Invitrogen) and Click-IT cell reaction buffer kit (Invitrogen #C10269). Images were acquired using an Axiovert 200M confocal microscope equipped with an LSM510 laser module (Zeiss).

### Immunoprecipitations

Extracts for immunoprecipitation were prepared using immunoprecipitation buffer (50 mM HEPES, pH 7.5, 150 mM NaCl, 1 mM EDTA, 2.5 mM EGTA, 10% glycerol, 0.1% Tween) with protease inhibitors (1% aprotinin, 5 μg ml^−1^ leupeptin, 1 mM PMSF), phosphatase inhibitors (50 mM NaF; β-glycerophosphate; 0.5 μM Calyculin A) and 1 mM DTT. Endogenous immuniprecipitation was performed with Cyclin F antibody coupled to protein A beads and immunoprecipitation of ectopically expressed FLAG and HA proteins was performed with FLAG beads (Sigma, A2220) and HA beads (Sigma, A2095), respectively. Beads were washed in immunoprecipitation buffer and incubated with the lysate for 2 h at 4 °C on a rotator. The beads were washed five times in immunoprecipitation buffer followed by elution of bound proteins.

### Purification of recombinant proteins and binding assays

GST, WT and dl93–100 mutant GST-CDC6 fragments (amino acids 1–362) were expressed in *Escherichia coli* Rosetta (DE3) cells. Protein expression was induced with 0.2 mM IPTG for 4 h at 25 °C. Cells were lysed by sonication in lysis buffer (20 mM Tris, pH 8, 200 mM NaCl, 0.5% NP40) with protease inhibitors (1% aprotinin, 5 μg ml^−1^ leupeptin, 1 mM PMSF). Lysed cells were spun at 21,000*g* for 30 min and GST, WT and dl93–100 mutant GST-CDC6 (amino acids 1–362) were bound to glutathione-sepharose beads (Amersham, 17-0756-01) for 1 h at 4 °C. Beads were washed four times and resuspended in 500 μl lysis buffer. *In vitro* translated WT and MR/AA mutant Cyclin F, respectively, were allowed to bind for 2 h at 20 °C. After binding, the beads were washed four times in lysis buffer with protease inhibitors, and samples were eluted in 40 μl SDS sample buffer, separated on a SDS–polyacrylamide gel and analysed by Coomassie staining and autoradiography.

### *In vitro* transcription and translation

WT and MR/AA mutant Cyclin F were obtained by *in vitro* transcription and translation with the TNT-coupled reticulocyte lysate system (Promega, L1170) in the presence of L-[35S]methionine-cysteine following the instructions of the manufacturer.

### Pulsed-field gel electrophoresis

For directly assessing cellular DNA breaks, 10^6^ cells were collected after indicated treatment and casted into agarose inserts (plugs) before incubation in proteinase K buffer (0.5 M EDTA; 1% N-laurylsarcosyl; 1 mg ml^−1^ proteinase K) at 50 °C for 24 h. Plugs were washed four times in TE buffer (10 mM TRIS; 50 mM EDTA) and loaded onto a 1% agarose gel (pulsed-field grade, Bio-Rad) and separated by pulse field gel electrophoresis (PFGE) apparatus for 20 h at 120° angle, 60–240 s switch time, 4 V cm^−1^ (CHEF DR III, Bio-Rad).

### *In vitro* ubiquitylation assay

Briefly, FLAG-tagged WT Cyclin F or FLAG-tagged Cyclin F(LP/AA) were transfected into HEK-293T cells. Anti-Flag M2 agarose beads were used to immunoprecipitate the SCF^Cyclin F^ complex. The beads were washed four times in lysis buffer and twice in ubiquitylation reaction buffer (10 mM Tris-HCl, pH 7.5, 100 mM NaCl, 5 mM MgCl_2_ and 1 mM DTT). Beads were then used for an *in vitro* ubiquitylation assays, which were performed in a volume of 30 μl, containing 2 mM ATP, 0.5 μM E1 (Boston Biochem), 10 ng μl^−1^ Ubch3, 10 ng μl^−1^ Ubch5c, 1 μM ubiquitin aldehyde and 3 μg μl^−1^ ubiquitin (Sigma)^25^. The reactions were incubated at 30 °C for 2 h and analysed by immunoblotting with antibodies to CDC6.

### Senescence staining

β-Galactosidase staining was performed with the Senescence β-Galactosidase Staining Kit (Cell Signaling, #9860) according to the manufacturer's instructions.

## Additional information

**How to cite this article:** Walter, D. *et al*. SCF^Cyclin F^-dependent degradation of CDC6 suppresses DNA re-replication. *Nat. Commun.* 7:10530 doi: 10.1038/ncomms10530 (2016).

## Supplementary Material

Supplementary Information Supplementary Figures 1-7
