# Supplementary material for: SCFCyclin F-dependent degradation of CDC6 suppresses DNA re-replication
Source: Nat Commun. 2016 Jan 28;7:10530. doi: 10.1038/ncomms10530 (PMC4738361; doi:10.1038/ncomms10530)
Supplement: Supplementary Information — Supplementary Figures 1-7 [file ncomms10530-s1.pdf]

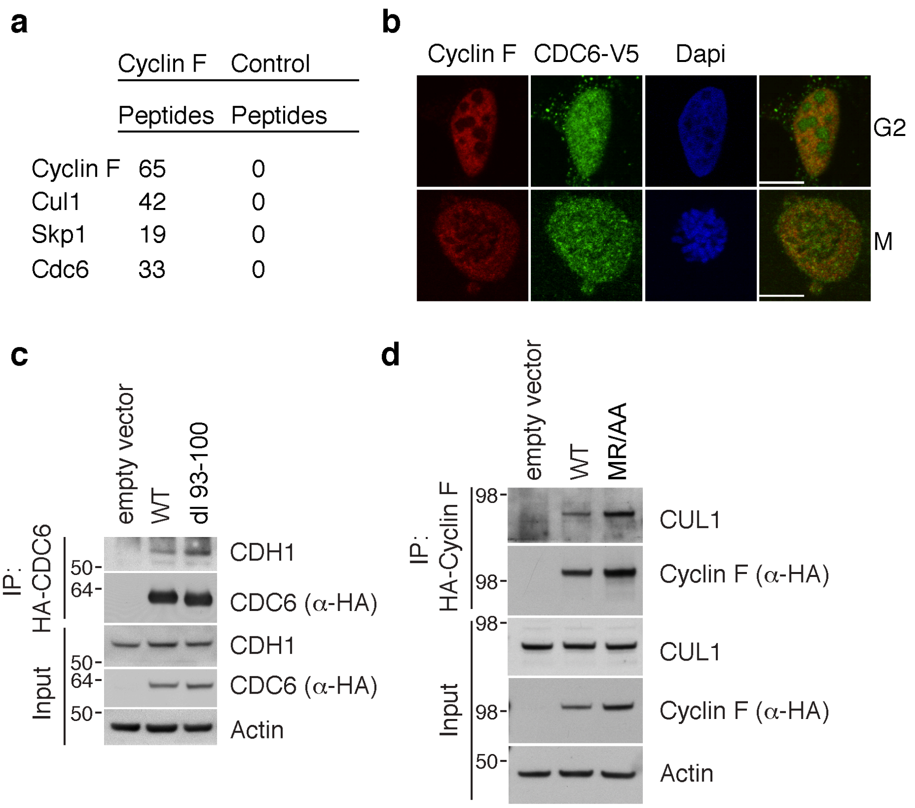

Supplementary Figure 1. Cyclin F and CDC6 interact and colocalize in G2 and M. **(a)** Whole-cell extracts of U2OS cells stably expressing FLAG-HA-Cyclin F were immunoprecipitated (IP) with anti-FLAG resin, and immunoprecipitates were analyzed by masspectrometry. **(b)** U2OS cells stably expressing CDC6-V5 were synchronized at G1/S by using a double-thymidine block before release into fresh medium for 10 hours (G2) and 13 hours (M), respectively. Cells were fixed and incubated with an anti-Cyclin F antibody (red) and anti-V5 antibody (green). DNA was stained with DAPI. Scale bar, 10 µM. **(c)** HEK-293T cells were transfected with an empty vector, HA-tagged wild-type CDC6 (WT) or HA-tagged mutant CDC6 (dl 93-100). Whole-cell extracts were immunoprecipitated (IP) with anti-HA resin, and immunoprecipitates were analyzed by immunoblotting as indicated. **(d)** HEK-293T cells were transfected with an empty vector, HA-tagged wild-type Cyclin F (WT), or HA-tagged mutant Cyclin F(MR/AA). Whole-cell extracts were immunoprecipitated (IP) with anti-HA resin, and immunoprecipitates were analyzed by immunoblotting as indicated.

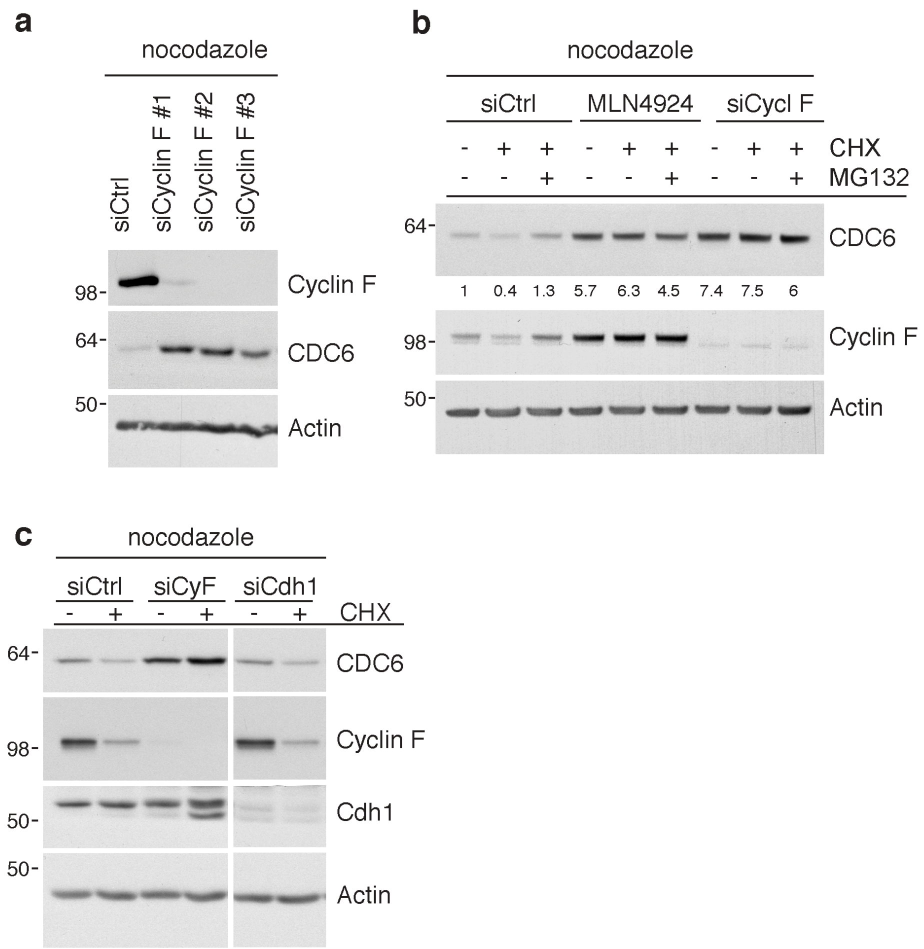

Supplementary Figure 2. CDC6 protein instability during mitosis depends on SCF$^{\text{Cyclin F}}$ but not on the Anaphase-Promoting Complex (APC).
(a) U2OS cells were transfected with control (siCtrl) or Cyclin F (siCycl F) siRNAs. After 48h cells were treated with nocodazole for an additional 5 hours and whole-cell extracts of cells obtained by mitotic shake-off were analyzed by immunoblotting as indicated. **(b)** U2OS cells were transfected with control (siCtrl) or Cyclin F (siCycl F) siRNA. After 48h cells were treated with nocodazole for an additional 5 hours and the protein stability in cells obtained by mitotic shake-off was assessed by CHX treatment for 90 minutes and immunoblotting. SCF complex E3 ligase inhibitor MLN4924 was added to mitotic shake-off cells for 90 minutes as indicated. Quantification of immunoblots of CDC6 is indicated. Individual lanes were quantified using ImageJ software and normalized to Actin loading control. **(c)** U2OS cells were transfected with control (siCtrl), Cyclin F (siCycl F), Cdh1 siRNA. After 48hrs cells were treated with nocodazole for an additional 5 hrs and the protein stability in cells obtained by mitotic shake-off was assessed by CHX treatment for 90 minutes and immunoblotting.

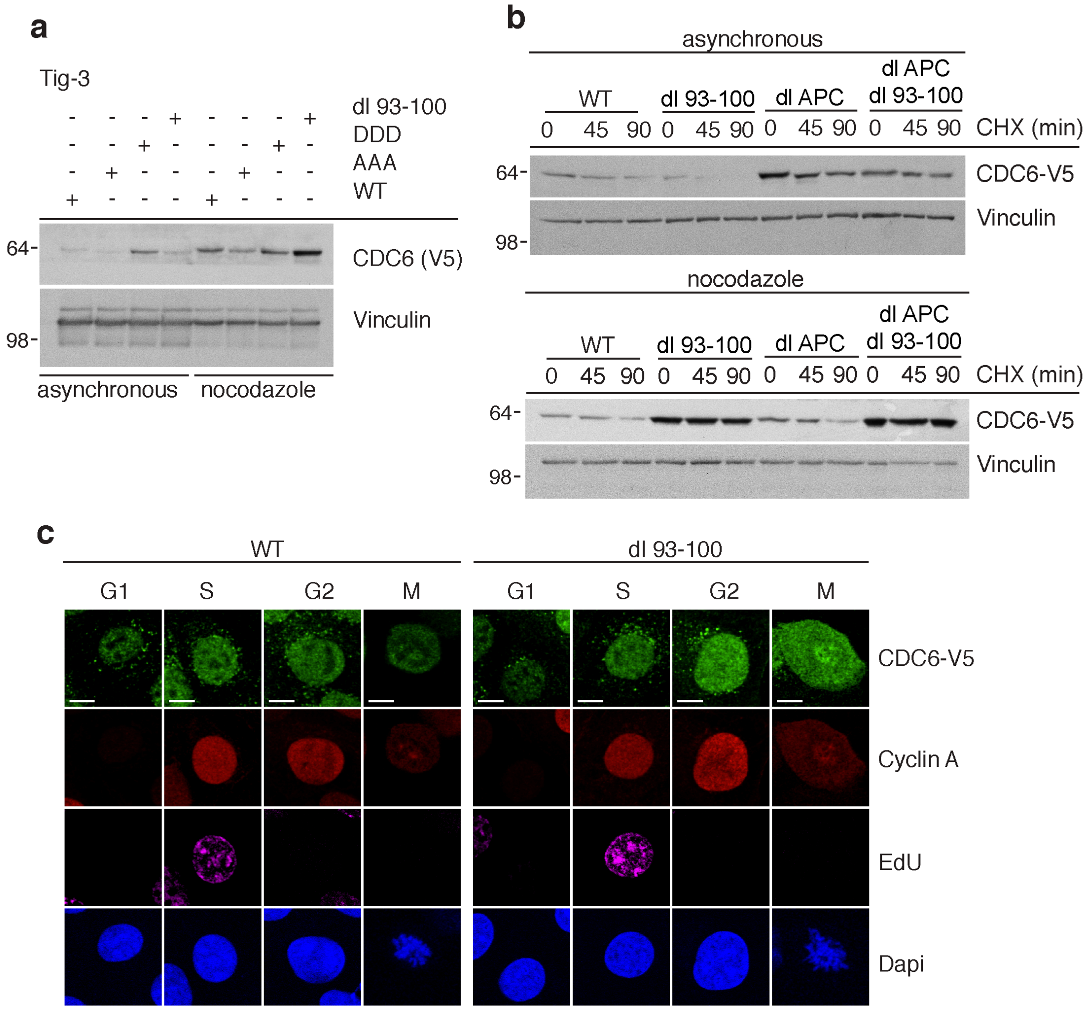

Supplementary Figure 3. (a) CDC6 protein instability during mitosis depends on the Cyclin binding motif but not on CDK phosphorylation and the Anaphase-Promoting Complex (APC). Tig-3 fibroblasts stably expressing either V5-tagged wild-type CDC6 (WT) or V5-tagged mutant CDC6 (AAA, DDD or dl 93-100, respectively) were treated with nocodazole for 5 hours as indicated and protein levels of WT and mutant CDC6 were assessed in asynchronous growing cells as well as in mitotic cells obtained by mitotic shake-off as indicated. **(b)** Protein stability of V5 tagged wild type CDC6 and V5-tagged mutant CDC6 (dl 93-100, dl APC, dl 93-100 dl APC) was assessed in asynchronous growing cells as well as in mitotic cells obtained by nocodazole treatment for 5 hours and mitotic shake-off as indicated. **(c)** U2OS cells stably expressing either V5-tagged wild-type CDC6 (WT) or V5-tagged mutant CDC6 (dl 93-100) were pulse-labeled with EdU for 30 min. Cells were fixed and subjected to Click-it reaction, Cyclin A and V5 staining before analysis by immunofluorescence. DNA was stained with DAPI. Scale bar, 10 μM.

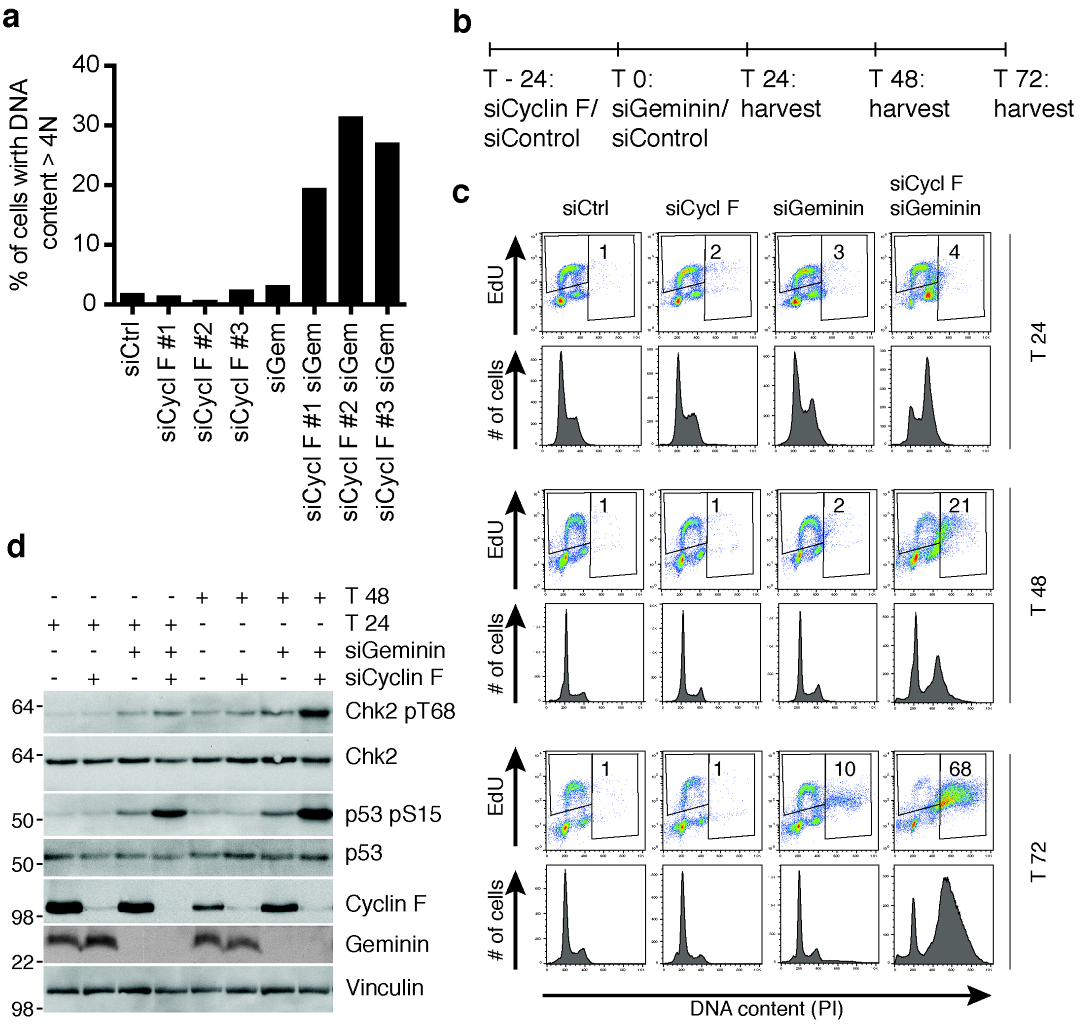

Supplementary Figure 4. Cyclin F suppresses re-replication.
(a) U2OS cells were first transfected with siRNA targeting Cyclin F as indicated. After 24 hours, transfection with siRNA targeting Geminin was performed as indicated. Cells were collected 72 hours after the first transfection and DNA content was monitored by flow cytometric analysis. Percentages of cells with DNA content > 4N are shown. **(b)** Schematic shows experimental setup for (c) and (d). **(c)** U2OS cells were first transfected with siRNA targeting Cyclin F as indicated. After 24 hours, transfection with siRNA targeting Geminin was performed as indicated in (b). Cells were collected at the indicated time points after pulse-labeling with EdU for 1 hr. Cells were subjected to Click-it reaction and PI staining before analysis by flow cytometry. **(d)** U2OS cells were transfected with siRNA as in (c) and collected at indicated time-points. Whole-cell extracts were analyzed by immunoblotting as indicated.

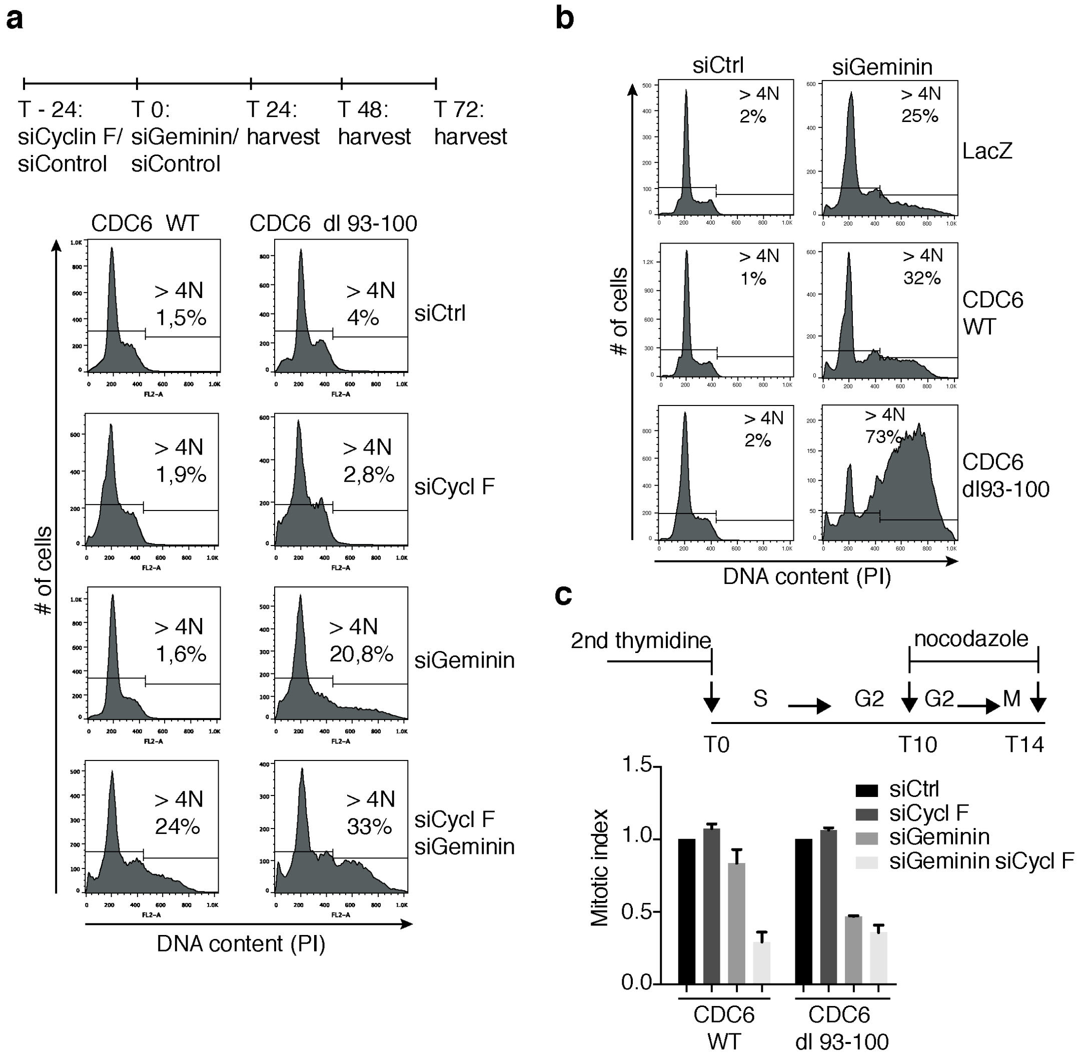

Supplementary Figure 5. Re-replication and impaired mitotic entry due to a lack of Cyclin F mediated CDC6 inhibition. **(a)** Schematic shows experimental setup. U2OS cells stably expressing V5 tagged wild-type CDC6 and V5 tagged mutant CDC6 (dl 93-100) were first transfected with Cyclin F and control siRNA, respectively. After 24 hours, cells were transfection with Geminin and control siRNA, respectively. Cells were collected 72 hours after the first transfection and DNA content was monitored by flow cytometric analysis. Percentages of cells with DNA content > 4N is indicated. **(b)** U2OS cells stably expressing V5 tagged wild-type CDC6, V5 tagged mutant CDC6 (dl 93-100) and LacZ, respectively, were transfected with Geminin and control siRNA as indicated. Cells were collected 48 hours after the transfection and DNA content was monitored by flow cytometric analysis. **(c)** Schematic shows experimental setup including a double thymidine synchronization and nocodazole treatment. U2OS cells stably expressing either V5-tagged wild-type CDC6 (WT) or V5-tagged mutant CDC6 (dl 93-100) were transfected with siRNAs targeting Cyclin F and Geminin in the first and second thymidine block, respectively. At the indicated time-points cells were given nocodazole for 4 hours to trap cells in mitosis. Cells were collected at indicated time-points and DNA content and mitotic MPM2 phosphorylation (p-MPM2) was monitored by flow cytometric analysis. The bar chart shows the mitotic index analyzed by p-MPM2 and normalized to control depletion (siCtrl). Error bars represent s.d. (n=3).

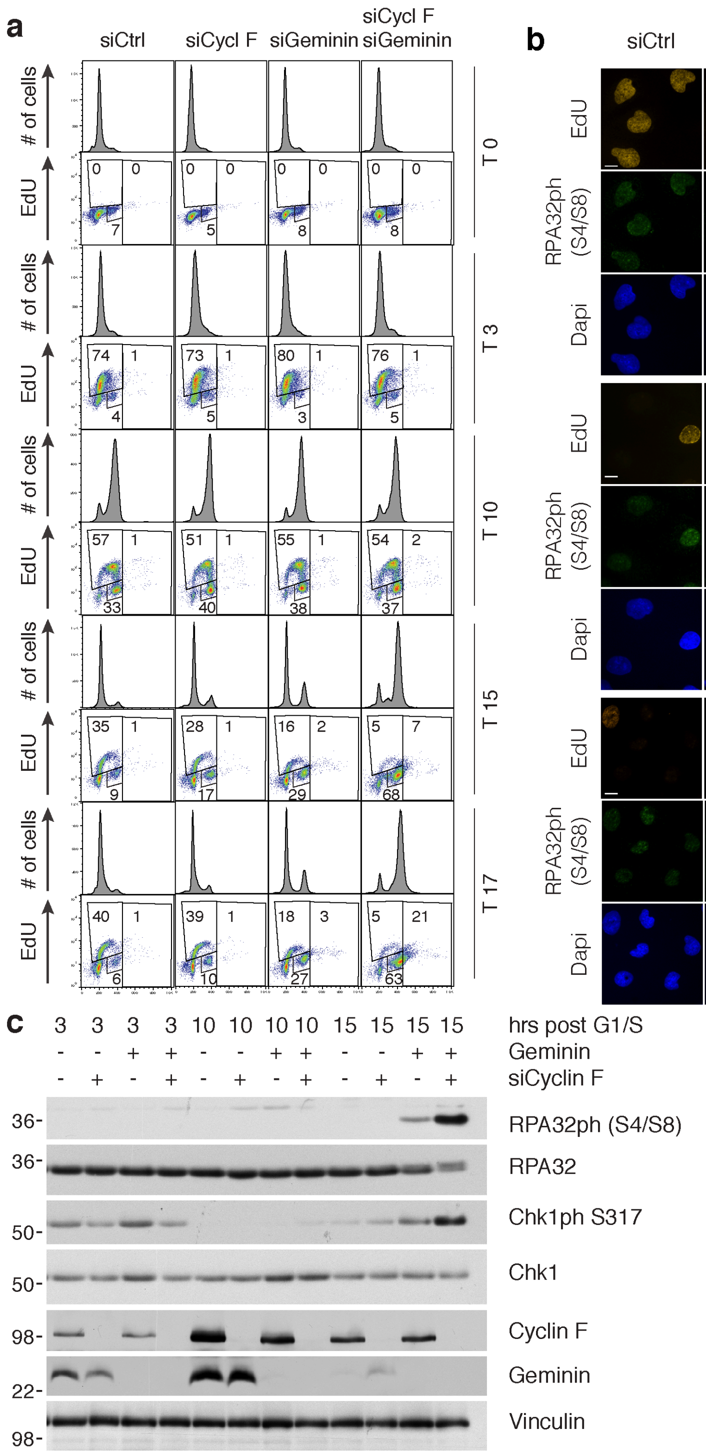

Supplementary Figure 6. Cyclin F mediated suppression of re-replication occurs during G2 phase. **(a)** U2OS cells were synchronized by double thymidine synchronization. Cells were transfected with siRNAs targeting Cyclin F and Geminin in the first and second thymidine block, respectively. Cells were collected at the indicated time points after pulse-labeling with EdU for 30 minutes. Cells were subjected to Click-it reaction and PI staining before analysis by flow cytometry. Percentages of cells in respective gates are indicated **(b)** U2OS cells were synchronized and transfected with siRNA as in (a). Cells were pulse-labeled with EdU for 30 min, fixed and subjected to Click-it reaction and RPA32 S4/S8 phosphorylation specific staining before analysis by immunofluorescence. DNA was stained with DAPI. Scale bar, 10 µM. **(c)** U2OS cells were synchronized and transfected with siRNA as in (a) and collected at indicated time-points. Whole-cell extracts were analyzed by immunoblotting as indicated.

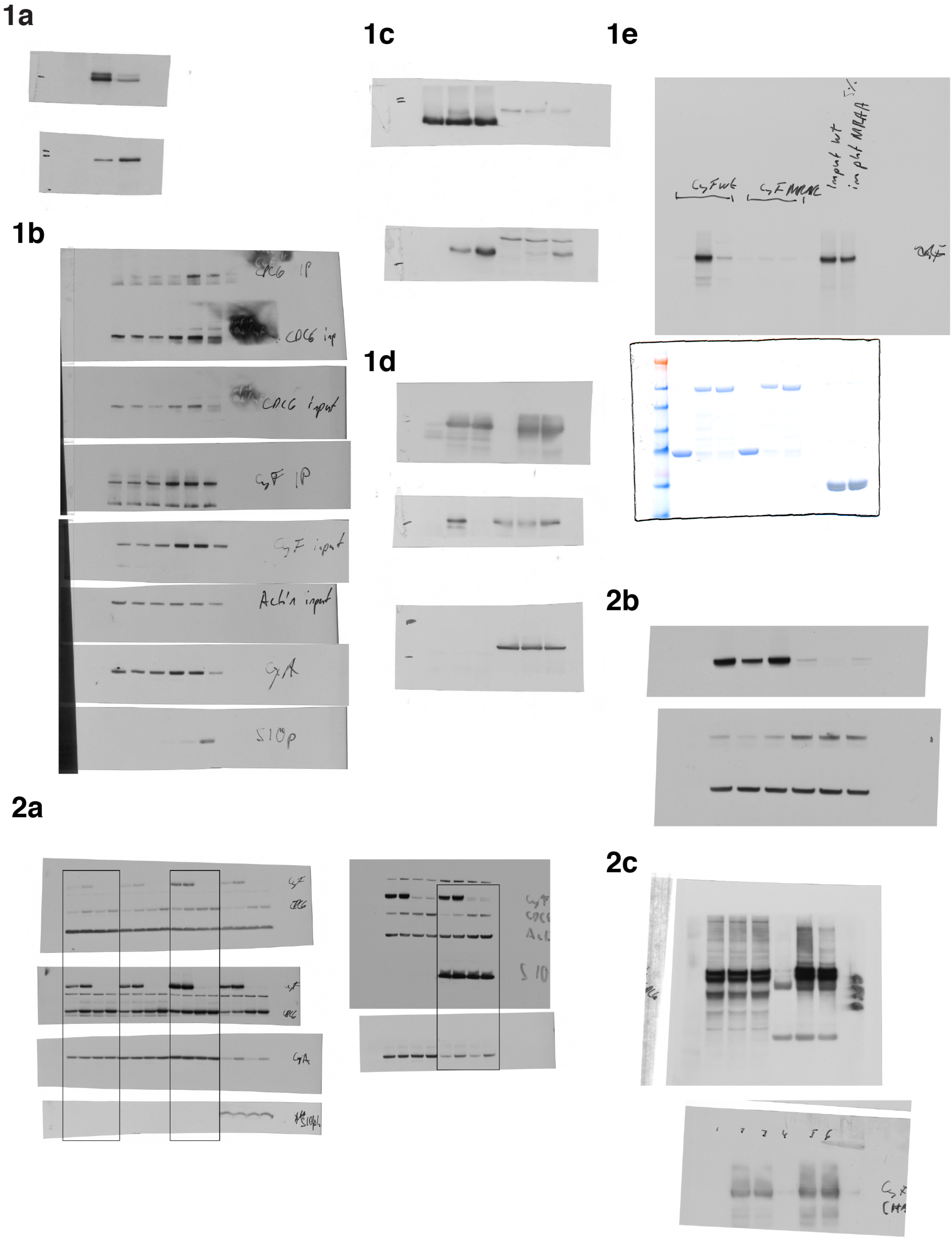

Supplementary Fig. 7: Uncropped images

3a

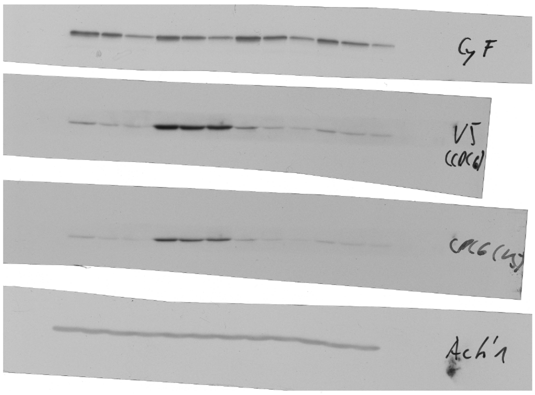

3b

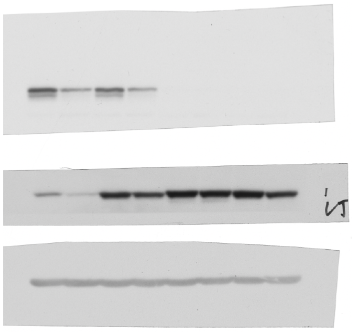

3c

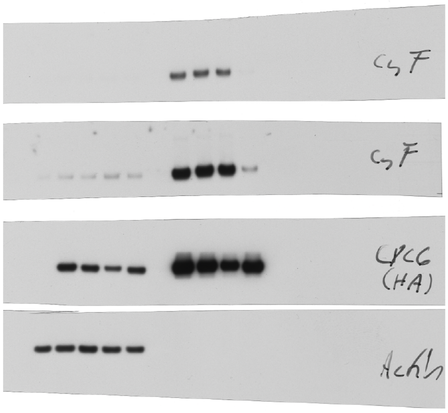

4b

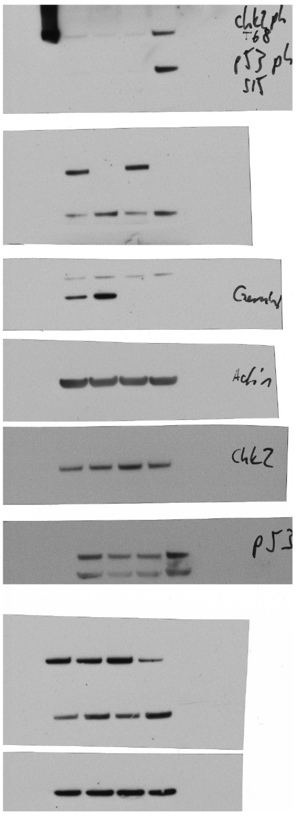

4d

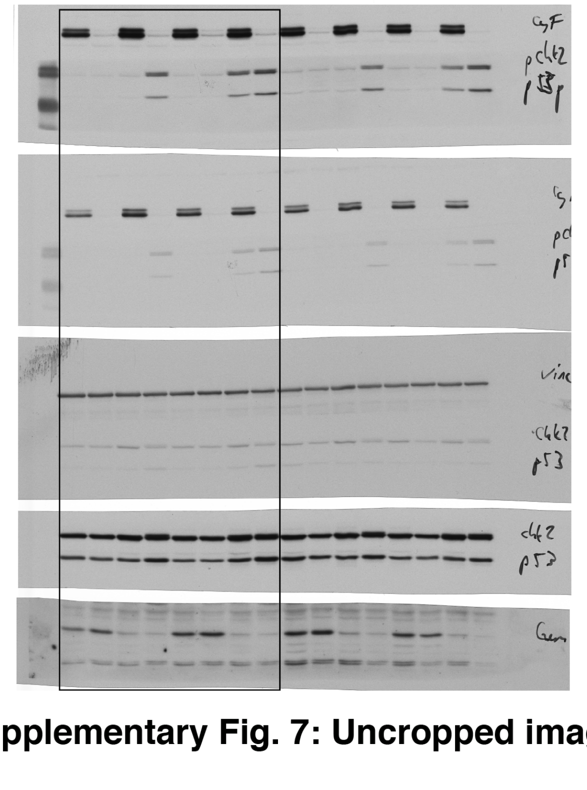

Supplementary Fig. 7: Uncropped images
